# Consequences of EMT-Driven Changes in the Immune Microenvironment of Breast Cancer and Therapeutic Response of Cancer Cells

**DOI:** 10.3390/jcm8050642

**Published:** 2019-05-09

**Authors:** Snahlata Singh, Rumela Chakrabarti

**Affiliations:** Department of Biomedical Sciences, School of Veterinary Medicine, University of Pennsylvania, Philadelphia, PA 19104, USA; snsingh@vet.upenn.edu

**Keywords:** breast cancer, subtypes, EMT, TWIST, MMPs, immune cells, TME, therapy resistance

## Abstract

Epithelial-to-mesenchymal transition (EMT) is a process through which epithelial cells lose their epithelial characteristics and cell–cell contact, thus increasing their invasive potential. In addition to its well-known roles in embryonic development, wound healing, and regeneration, EMT plays an important role in tumor progression and metastatic invasion. In breast cancer, EMT both increases the migratory capacity and invasive potential of tumor cells, and initiates protumorigenic alterations in the tumor microenvironment (TME). In particular, recent evidence has linked increased expression of EMT markers such as TWIST1 and MMPs in breast tumors with increased immune infiltration in the TME. These immune cells then provide cues that promote immune evasion by tumor cells, which is associated with enhanced tumor progression and metastasis. In the current review, we will summarize the current knowledge of the role of EMT in the biology of different subtypes of breast cancer. We will further explore the correlation between genetic switches leading to EMT and EMT-induced alterations within the TME that drive tumor growth and metastasis, as well as their possible effect on therapeutic response in breast cancer.

## 1. Introduction

Breast cancer is a highly complex disease that has been classified into several subtypes based on morphological, immunohistochemical, and phenotypic characteristics of the tumor. The most commonly used classification is based on the presence or absence of hormone receptors. Breast cancers expressing estrogen (ER), progesterone (PR), and herceptin (HER2) receptors are termed hormone receptive while those that lack all three receptors are classified as hormone refractory or triple-negative breast cancer (TNBC) [1,2]. Such heterogeneity complicates choice of treatment options and highlights the critical need to study breast cancer in a subtype-specific manner. 

Like other cancers, breast cancer is initiated by transformation of normal cells to cancerous ones. Following this transformation, epithelial-to-mesenchymal transition (EMT) plays an important role in enabling epithelial cells to acquire mesenchymal features and gain invasive potential [3,4,5], thereby driving cancer progression. During EMT, epithelial cells lose polarity and adhesive junctions that maintain cell–cell contact and undergo transformation to mesenchymal cells. Conversely, during mesenchymal-to-epithelial transformation (MET), tumor cells reacquire their epithelial characteristics and obtain cell–cell contact. MET is an essential step for tumor cells during colonization at the metastatic site [6,7,8]. EMT drives many developmental processes and is frequently observed in cancers, including breast cancer. EMT in the early stages of carcinogenesis is brought about by a switch in expression patterns of crucial genes, thereby initiating a cascade of cellular, molecular, and morphological changes in cells [3,4,5]. In addition to the dramatic effect of EMT on tumor cells, it brings a massive change in the dynamic landscape of the tumor microenvironment (TME). At the early stages of transformation, cytokines/chemokines secreted from tumor cells attract various stromal and immune cells to the TME [9,10]. These immune cells in turn provide a niche that facilitates tumor progression, invasion, and metastasis. Studies in the last decade have shown that immune cells in the TME determine the clinical outcome of the disease as well as the response of the tumor to chemo and immune therapy [11,12,13,14,15].

In this review, we will summarize the changes in gene expression during EMT leading to recruitment of immune cells in the TME that in turn facilitate progression, invasion, and metastasis of breast cancer. As breast cancer is notoriously heterogeneous and therapeutic regimen is decided according to the breast cancer subtype, we will focus on the role of EMT in different subtypes of breast cancer. We will also compile findings from studies describing how EMT-mediated changes in the immune landscape of the TME determine the therapeutic response of tumors.

## 2. Breast Cancer Subtypes and Their Association with EMT

As per the most recent molecular classification, breast cancer can be divided into the following subtypes: luminal A and B, HER2 positive, basal-like, and claudin-low. Luminal A and B breast cancers are generally ERα-positive. Luminal B tumors show higher expression of Ki67 and are therefore highly proliferative and associated with a worse prognosis [16,17]. HER2-positive tumors express the oncogene ERBB2 on their membrane [18,19]. Basal-like tumors show high expression of basal cell markers and basal cytokeratins [20,21]. Claudin-low tumors are high in stem-cell-associated processes and display high expression of genes involved in EMT [22,23]. Basal-like and claudin-low subtypes usually lack all of the characterized hormone receptors such as ER, PR, and HER2 and are categorized as triple-negative breast cancer (TNBC). 

Breast cancer cells arise from mammary epithelial cells that undergo various transcriptional, morphological, and biochemical changes, including EMT, that contribute to tumorigenesis. Normal mammary epithelial cells undergo EMT, a process that occurs in three distinctive phases, each bearing a distinct cassette of EMT-activating transcription factors (TFs). In the first phase, cells lose their polarity and acquire mesenchymal markers such as vimentin and fibronectin. After morphological changes, a switch in gene expression from epithelial-expressed E-CADHERIN to mesenchymal-expressed N-CADHERIN occurs that is mediated by ZEB1 and SNAIL and is maintained by GOOSECOID and FOXC2. During the third phase of EMT, mesenchymal cells acquire phenotypic and functional cancer stem cell (CSC) properties (CD44^high^CD24^low^, invasive, and tumorsphere-forming abilities) [24]. Acquisition of mesenchymal properties by tumor cells is associated with an upregulation of EMT transcriptional inducers such as TWIST1/2, SNAI2/SLUG, and ZEB1 [25,26,27,28]. Physiological regulators such as Notch receptors and ligands, along with Wnt ligands, can induce EMT in mammary epithelial cells. Notch and Wnt factors are also important for different steps of breast cancer initiation and progression [29,30,31,32,33]. In addition, EMT in normal mammary epithelial cells can be induced by overexpression of the apoptosis regulator B-cell lymphoma/leukemia gene 2 (BCL2), highlighting a novel role of BCL2 in EMT [34]. Sarrio et al. showed that the nontumorigenic basal cell lines MCF10A, MCF10-2A, and MCF12A contain an epithelial subpopulation which is epithelial cell adhesion molecule (EPCAM)-positive and spontaneously generates EPCAM negative mesenchymal cells through EMT that exhibit CSC (CD44^high^CD24^low^) properties, as they are capable of forming tumorspheres and have increased invasive potential [25]. Consequently, it was suggested that EMT can increase the heterogeneity of the stem cell population in normal breast tissue, with a subset of epithelial cells displaying normal stem-cell-like features and a mesenchymal subset exhibiting CSC features that may contribute to tumor initiation and early dissemination. 

### 2.1. Luminal A and B Breast Cancers and EMT

Tumors of the luminal A subtype are observed in the majority of breast cancer patients and both luminal subtypes A and B express ERα. Although estrogen signaling is necessary for breast tumor growth [35], the ERα signaling pathway can inhibit EMT [36,37], raising the intriguing possibility that ERα expression could be responsible for the better prognosis of luminal A and B patients as compared to TNBC patients. Mechanistically, Ye et al. showed that ERα prevents EMT through repression of SLUG, either by directly decreasing its transcription or by repressing the nuclear coreceptor which binds to the SLUG promoter, thereby increasing expression of E-CADHERIN [36]. In a similar study, Wang et al. reported that ERα inhibits EMT by inhibiting RELB-dependent BCL2 expression in luminal breast cancer cell lines [37]. Alternatively, another study showed that ERα suppresses BM1 and therefore promotes stemness and EMT in breast cancer cells [38]. It will be interesting to determine how ERα signaling promotes these distinct functions in breast cancer cells and how these events are regulated in future experiments.

Extrinsic factors, like the multipotent cytokine transforming growth factor β (TGF-β) stimulates EMT in breast cancers [39] and mechanistically, TGF-β stimulation is associated with upregulation of SNAIL, TWIST, ZEB1/2 in luminal A and B breast cancer cell lines [40,41,42]. TGF-β-induced EMT activates EGFR-, IGF1R-, and MAPK-dependent ERα signaling and promotes antiestrogen resistance [43]. Similar to the TGF-β pathway, the MEK–ERK pathway represses ESE1, a member of the ETS transcription factor family, resulting in upregulation of ETS1-regulated ZEBs. Therefore, activation of MEK–ERK positively correlates with an EMT phenotype in the luminal subtype of breast cancer [44,45]. Finally, in the luminal cancer cell line MCF7, VEGFR expression positively correlates with expression of SNAIL and N-CADHERIN, key regulators of EMT [46]. These studies suggest that while ERα can prevent EMT, environmental stimuli such as TGF-β and activators of the MEK–ERK pathway can promote EMT in luminal cancer, indicating that the final outcome depends on a balance between these pathways.

### 2.2. HER2-Positive Breast Cancer and EMT

Similar to luminal subtypes of breast cancer, HER2-positive breast cancers also undergo TGF-β-dependent EMT. Chihara et al. showed that the TGF-β–SMAD3 pathway is critical for EMT in HER2-positive cancers [47]. Analysis of signaling pathways influencing TGF-β expression in HER2-positive tumors revealed that silencing of AXL, a receptor tyrosine kinase that correlates with poor survival in HER2-positive patients, in a patient-derived xenograft reduces TGF-β, thereby impairing invasion [48]. Notably, HER2 directly regulates the production of TGF-β and activation of TGF-β/SMAD3 signaling [49]. This HER2/EGFR signaling controls the switch from a cell proliferative function for TGF-β to promotion of cell migration [50], therefore making it a central player in the functional consequences of EMT in HER2-positive tumors.

Along with TGF-β/SMAD3 signaling, upregulation of transcription factors SLUG and TWIST1 plays an important role in EMT in this breast cancer subtype. In HER2-positive breast cancer cell lines, MDA-MB-453 and BT474, Carpenter et al. showed that activation of AKT signaling upregulates SLUG expression [51]. However, clinical studies in which patients were categorized based on surface marker expressions showed that HER2-positive patients do not exhibit strong nuclear expression of SLUG [52], highlighting the need for further careful investigation. TWIST1, a known regulator of EMT, is highly phosphorylated on Serine 68 residue in HER2-positive invasive ductal carcinomas, thereby stabilizing the protein and promoting breast cancer invasiveness [53]. In addition, overexpression of HER2 in MCF7 luminal cells increased the expression of breast tumor kinase (Btk)/protein tyrosine kinase 6 (PTK-6) receptors, thereby augmenting EMT and invasive potential [54].

These studies collectively establish the role of TGF-β-associated pathways along with TWIST and SLUG genes in mediating EMT in HER2-positive breast cancer. Further studies are needed to identify novel pathways and mechanisms behind EMT in HER2-positive breast cancer.

### 2.3. TNBC or Basal-Like, Claudin-Low Breast Cancer and EMT

TNBCs are the most aggressive subtype of breast cancer, with limited therapeutic options due to their lack of hormone-responsive receptors. Based on their molecular characteristics, TNBC can be further divided into different subtypes such as PAM50, Vanderbilt, Baylor, and French [55]. In addition, TNBCs can be classified into four categories; basal-like, mesenchymal, immunomodulatory, and luminal androgen receptor (AR)-positive subtypes [56]. In the basal-like subtype of TNBC, cell cycle and DNA damage response pathways are highly activated, so these tumors are often treated with platinum drugs and ADP-ribose polymerase (PARP) inhibitors that target these pathways [57,58,59]. Genome analysis of mesenchymal TNBC tumors shows high expression of gene clusters involved in growth factor signaling, such as PI3K/AKT, along with an increase in EMT gene signatures. Accordingly, these tumors are susceptible to mTOR inhibitors and eribulin mesylate, which is an inhibitor of EMT [60]. Immunomodulatory TNBCs are enriched in gene pathways related to immune cell signaling associated with immune cell recruitment, as well as signal transduction such as NFκB and JAK/STAT pathways. Thus, in patients with immunomodulatory TNBC, immune checkpoint inhibitors have yielded promising results [61,62]. Luminal androgen receptor (AR)-positive subtype tumors have high levels of androgen-associated signaling and are therefore responsive to androgen receptor blockade [63]. 

EMT-related factors that have been widely described in TNBC are Notch and Hedgehog, TGF-β, and WNTs. High Notch expression in tumor samples from TNBC patients correlates with poor survival [64]. NUMB, an evolutionary conserved protein important for cell fate determination, antagonizes Notch signaling to prevent EMT in TNBC [65]. JAGGED1, a Notch ligand, can activate Notch signaling to induce EMT through upregulation of SLUG, which in turn represses the expression of E-CADHERIN [66]. Another recent study has shown that the Notch receptor NOTCH3 is important for TNBC breast cancer growth [67]. Moreover, reports show that NOTCH1 and NOTCH4 represent potential biomarkers in TNBC due to their high expression [68]. However, their connection to EMT in breast cancer is not very clear and further studies are needed to confirm the involvement of Notch signaling at the level of each receptor and ligands for EMT in TNBC. Like the Notch pathway, the Hedgehog pathway is crucial for embryonic development and stem cell renewal, and has also been associated with EMT in breast cancer. Hedgehog signaling activates three glioma-associated oncogenes, GLI1, 2, and 3. By employing a high-throughput screen, Colavito et al. have identified GLI1 as a critical determinant of EMT in breast cancer cell lines [69]. Activation of GLI1 is also associated with hypoxia-induced EMT and invasive potential of MDA-MB-231 TNBC cells [70]. Other Hedgehog signaling factors like SHH, PTCH1, and GLI2 are overexpressed in breast cancer, but their connection to EMT is not well established in breast cancer [71,72].

As in other types of breast cancer, TGF-β-mediated regulation of N-CADHERIN, BCL2, and CYCLIN D1 determines EMT and stemness in MDA-MB-231 TNBC cells [73]. In addition, musculoaponeurotic fibrosarcoma (MAF) oncogene family protein K (MAFK) induces EMT in a TGF-β-dependent manner in TNBC cell lines [74]. These studies suggest that TGF-β could be a universal master regulator of EMT in tumor cells. The functional importance of EMT in tumor progression was further demonstrated by addition of selective inhibitors of inducible nitric oxide synthase (iNOS), an enzyme associated with poor prognosis in TNBC patients. These inhibitors limited migration and self-renewal properties of TNBC cells along with reducing the levels of EMT transcription factors such as SNAIL, SLUG, TWIST1, and ZEB1 [75]. Interestingly, targeting β3 integrins using nanoparticles-based siRNA inhibited EMT and metastasis in TNBC tumors by attenuating TGF-β signaling [76]. Thus, TGF-β is connected to EMT either directly or indirectly promoting breast cancer progression.

Aberrant Wnt signaling is a characteristic of TNBC, with both canonical and noncanonical pathways implicated in TNBC tumorigenesis and metastasis [77,78]. Enrichment of Wnt/β-catenin signaling is evident in TNBC and is associated with poor clinical outcome within this subtype [78]. Earlier studies from our group showed that ΔNP63, a transcription factor, upregulates FZD7, a Wnt receptor, thereby increasing Wnt signaling and EMT in normal mammary stem cells and basal subtype of breast cancer [77]. Along with Wnt activators, GSK3β, a canonical Wnt pathway inhibitor, plays an important role in EMT in TNBC cells. A recent study shows that GSK3β is a potential therapeutic target for TNBCs and suggests that GSK3β inhibitors could serve as selective inhibitors of EMT and CSC function in the treatment of a subset of aggressive TNBC with more mesenchymal cells [79]. Another recent study shows that WNT10B, a noncanonical ligand, is important for EMT and CSC-like phenotypes in TNBC in a preclinical mouse model [80]. Together, these studies highlight that EMT is an integral part of multiple subsets of breast cancer. It can be regulated by diverse cell signaling mechanisms, and therapeutic targeting of EMT pathway may be beneficial, even in breast cancer subtypes that are notoriously treatment-resistant. We have summarized various genes and pathways responsible for EMT in different breast cancer subtypes in Table 1. 

## 3. EMT Shapes the TME 

Multiple studies demonstrate that EMT is associated with increased dissemination and metastasis of tumor cells to other organs [26,81,82]. In part, this is due to the ability of tumor cells undergoing EMT to modulate the TME. Dvorak H.F. in 1986 in his highly cited article, “Tumors: wounds that do not heal: Similarities between tumor stromal generation and wound healing”, explains how phenomena that occur within tumor stroma are similar to processes underway at a wound site [83]. Later studies by Coussens et al. suggested that precancerous cells are identified as a “wound” by mast cells [84], and similar to wounds, high numbers of platelets are found at sites of tumorigenesis [83,84]. Coussens and Hanahan went on to describe tumor growth as a biphasic event [85]. In the first phase, the body treats the tumor site as a wound and tumor growth is promoted by stromal cells. In the second phase, the tumor takes control of proinflammatory cytokines and shapes the TME to further support cancer growth and metastasis. Similar observations are seen in breast cancer where the tumor growth is aided by the TME and at the same time the TME confers proinvasive features to the tumor cells [86].

Based on these observations, it is critical to understand the transcriptional events within the tumor that have a subsequent impact on TME function, thereby influencing tumor progression. RUNX3, a member of the RUNX family of transcription factors, is frequently connected to breast cancer [87]. The immune suppressive role of RUNX3 has been reported in breast tumors via regulation of Tregs. A recent report found that RUNX3 binds to the promoter of FOXP3 and increases Treg population in the tumor microenvironment, which is associated with the progression of breast tumors [88]. However, RUNX3 has also been indicated as a tumor suppressor in breast cancer, which needs further careful evaluation [87]. Similar to RUNX3, the transcription factor GATA3 inhibits breast cancer progression and metastasis by altering the TME [89,90]. Furthermore, overexpression of members of the ETS family of transcription factors can promote increased numbers of immune cells in the TME to drive tumor progression. For example, complete deletion of ETS2 from epithelial and stromal cells in breast tumors leads to early hyperplastic growth and tumor formation by affecting MMP-3 and MMP-9 in macrophages in TME [91,92]. We have reported that ELF5, another member of the ETS family, suppresses EMT and metastasis of TNBC cells [93]. In an unpublished work from our lab, we have seen that loss of ELF5 in a preclinical TNBC mouse model not only enhances tumor growth and metastasis, but also leads to increased numbers of immune cells in the TME. Previously, we had shown that another transcription factor, ΔNP63, promotes stem cell activity in basal tumors [77] and that its expression positively correlates with EMT in basal tumors [77]. Recently, we showed that overexpression of ΔNP63 induces tumor cell production of CXCL2 and CCL22, chemokines responsible for recruitment of MDSCs and enhancing growth and metastasis of basal tumors [94]. P53, a tumor suppressor, regulates miRNAs to inhibit EMT and stem cells by regulation [95]. In a separate study, p53 levels were associated with increased numbers of lymphocytes in basal breast cancer [96]. These studies suggest that transcription factors intrinsic to tumors are important in shaping the TME. For a comprehensive understanding of how cancer cell intrinsic mechanisms such as transcription factors and other genes shape tumor immune microenvironment, please refer to the recent extensive review [97].

Preparation of tumor in premetastatic niches also involves modulation of the extracellular matrix (ECM). Matrix metalloproteinases (MMPs) are a group of 23 enzymes, 17 of which are secreted and 6 are membrane-bound. MMPs are implicated in modification of the ECM, leading to tumor development, migration, and invasion. MMP-3 or MMP-7 overexpression in the mammary epithelium generates premalignant lesions and spontaneous tumor formation [98,99]. On the contrary, MMP-11 knockout mice treated with the carcinogen 7,12-dimethylbenzanthracene (DMBA) develop fewer tumors than control [100]. While epithelial cells can produce MMPs that promote protumorigenic changes in the ECM, a few reports suggest that epithelial cells undergoing EMT can also give rise to myofibroblasts and stromal-like cells that are an essential part of tumor stroma [101,102]. These myofibroblasts produce additional MMPs to assist tumor growth and invasion [103,104,105]. 

In addition to modulating ECM at the site of tumor generation, epithelial cells undergoing EMT secrete soluble factors and cytokines to create an inflammatory environment for recruitment of lymphocytes, leucocytes, and other immune cells. Two of the well-studied cytokines produced by tumor cells are Interleukin 6 (IL-6) and IL-8. IL-6 is overexpressed in multiple cancers including breast cancer [106,107] and high expression levels correlate with poor clinical outcomes in cancer patients [108]. IL-6 promotes tumorigenesis in a cancer cell autonomous manner as well as by influencing the differentiation of immune cells [109,110], including B cells, T cells, and myeloid cells, and by promoting immunoglobulin production by B cells. Circulating IL-6 levels correlate with worsening prognosis in metastatic breast cancer patients and also correlate with the extent of the disease [111]. In breast cancer, IL-6 on tumor cells has been shown to induce EMT by repressing E-CADHERIN via STAT3 activation [112]. In another study involving multiple breast cancer subsets, IL-6 has been shown to increase cancer stem cell properties of tumor cells via EMT [113]. IL-6 levels also correlate to increased number of MDSCs, tumor-associated neutrophils (TANs), regulatory T cells (Tregs) in many cancers including breast cancer, suggesting that the consequent immune-suppressive environment contributes to cancer evasion [114]. Dominguez et al. reported that neutralization of IL-8 in TNBCs not only reduces their mesenchymalization but also reduces the number of polymorphonuclear MDSCs (PMN-MDSCs). This suggests that IL-8 both promotes EMT in tumors and recruits immune cells involved in creating an immunosuppressive TME for progression and metastasis of tumor cells [115]. Together, these studies suggest that soluble factors and chemokines secreted by epithelial cells undergoing EMT play a critical role in restructuring the ECM and immune landscape to support tumor proliferation, progression, and metastasis. 

## 4. Regulation of EMT by Immune Cells in TME

As detailed above, soluble factors released by cells undergoing EMT create an inflammatory milieu that promotes recruitment of immune cells to the site of tumorigenesis. These immune cells infiltrate the TME and assist tumor growth. In this subsection, we will highlight how different immune cells like macrophages, MDSCs, NK, and Tregs promote EMT and tumor progression in breast cancer.

Macrophages are monocytes that can be differentiated into M1 (antitumorigenic) and M2 (protumorigenic) phenotypes [116]. Recruitment of monocytes to the TME through stimuli such as CCL2/monocyte chemoattractant protein 1 (MCP-1) or colony-stimulating factor 1 (CSF-1) is well studied [117,118,119]. Stimulation of such monocytes with IL-4/IL-13, IL-10, or TGF-β leads to generation of M2 macrophages [120] or TAMs which facilitate tumor angiogenesis and immune suppression, invasion, and metastasis by limiting the ability of CD8^+^ cytotoxic T cells. Macrophages are thought to promote early dissemination of cancer, angiogenesis, and metastasis by enhancing CSC-like features in tumor cells through EMT [121,122]. Specifically, TAMs secrete proangiogenic factors such as VEGF, PDGF, TGF-β and MMPs, IL-6, and IL-1 to induce neovascularization and promote EMT [123,124,125]. Through modulation of the TME and ECM, TAMs provide a prometastatic environment for tumor cells. In ER-positive luminal cancer cells like MCF7 and T47D, secretory factors like MMP-9 promote invasive and migratory potential in cancer cells once they are cultured with macrophages [126]. Depletion of TAMs by anti-CSF1 antibody, which is a macrophage regulator, in a luminal breast cancer model leads to a reduction in tumor growth [127]. In this model, increased TAMs result in a TME rich in TGF-β, an inducer of EMT, and is associated with increased invasion by tumor cells. TAM numbers also correlate with EMT and low E-cadherin expression levels and can therefore be used as an unfavorable prognostic factor for TNBC [128]. These data suggest that TAMs may promote EMT in multiple breast subsets to promote tumor progression and metastasis. As such, defining the precise mechanisms regulating the differentiation of TAMs from infiltrating macrophages in breast cancer may provide crucial insight for therapeutic intervention.

Myeloid-derived suppressor cells (MDSCs) contribute to invasion and metastasis of cancer in multiple ways, but their primary action is through suppression of the antitumor immune response [129]. Myeloid cells infiltrating into the TME during initial stages of tumorigenesis differentiate into MDSCs in the chronic proinflammatory environment of the TME. Indeed, activated T cells secrete IFN-γ, which plays a crucial role in differentiation of MDSCs from myeloid cells [130,131]. These activated MDSCs express CD40 and PD-L1, which suppress the antitumor response of T cells [132,133]. Additionally, MDSCs produce Prostaglandins E2 that amplify MDSC populations in the TME [134]. Indoleamine 2,3-dioxygenase (IDO) is often expressed on tumor cells and are responsible for recruitment of MDSCs in creating an immune-suppressive environment [135] via regulatory T cells (TRegs) which produce kynurenine in several cancers like melanoma [136]. This suggests that therapeutic targeting of IDO could be one of the central regulators of immune suppression. Similar correlation between IDO and MDSC has been observed in metastatic breast cancer patients [137]. Future studies delineating the molecular mechanism of IDO-mediated recruitment of MDSCs in breast cancer may provide innovative therapeutic strategies.

In addition to the immune suppressive property of MDSCs, recent studies show a novel nonimmunologic function of MDSCs in increasing CSCs in breast cancer, which in turn makes the tumor cells more invasive and metastatic [138,139,140]. Our study showed that PMN MDSCs are higher in the basal subset of TNBC and are recruited in a ΔNP63-dependent manner [94]. In return, these MDSC secrete prometastatic factors that increase EMT gene signatures and CSC gene signatures in the TNBC cells, making them more invasive and metastatic. In another recent example using the 4T1 TNBC mouse model, it was shown that CXCR2^+^ MDSCs induces cancer cell EMT by IL-6 and these CXCR2^+^ MDSCs promotes T cell exhaustion, suggesting that CXCR2^+^ MDSCs may be a potential therapeutic target of TNBC [141]. Interestingly, MDSCs differentiate to tumor-associated macrophages in tumors, which are often more immune suppressive and support cancer stem cell properties. Together, MDSC and TAMs promote EMT and metastasis of breast cancer [142]. Thus, understanding the molecular mechanism of this differentiation step is integral to development of novel drugs targeting these immune-suppressive cells in breast cancer.

NK cells are classically known to induce antitumor immune responses [143,144]. However, multiple recent reports suggest that they may also promote tumor progression and metastasis in cancers in part by regulating EMT [145,146,147]. IL-18, present in the TME, can upregulate PD-1 expression on NK cells, resulting in an immune suppressive phenotype [148]. NK cells residing in tumors have a reduced antibody-dependent, cell-mediated cytotoxicity (ADCC) potential, thus limiting their antitumor activity [149,150]. Interestingly, tumor cells expressing Cell Adhesion Molecule 1 (CADM1), a cell adhesion molecule directly induced by the EMT-promoting TGF-β pathway [151], are susceptible to NK cell-mediated cytotoxicity [152]. In a cohort of breast cancer patients, CADM1 expression correlated with improved patient survival [153]. While these studies point towards a strong association between NK cell function and EMT in tumors, further investigation on their role in tumorigenesis is required. 

T cells are a critical regulatory factor in tumor biology. CD8+ cytotoxic T cells secrete antitumor cytokines such as TNFα and IFN-γ that restrict the growth and metastasis of tumors [154,155,156]. 

However, CD8+ T cells within the TME frequently exhibit an “exhausted” phenotype due to overexposure to tumor antigens and/or the presence of immune suppressive antigens on tumor cells. Exhausted T cells neither produce antitumor cytokines nor undergo proliferation, thus restricting their antitumor activities [157]. In addition, FoxP3+ Tregs help tumor cells grow and metastasize through production of protumorigenic cytokines and expression of immunomodulatory receptors that suppress immune response and facilitate tumor growth [158]. Moreover, Tregs promote β-catenin-mediated EMT during radiation-induced pulmonary fibrosis [159], however, the molecular mechanism is not clear. In this regard, in our unpublished study, we have observed high levels of Treg infiltration in a preclinical murine model of TNBC undergoing EMT. Our future studies will establish the molecular mechanisms behind the association of Tregs and EMT in TNBC. Together, these reports collectively highlight that immune cells in the TME recruited during early stages of EMT additionally assist tumor cells in their proliferation, invasion, and metastasis in breast cancer. 

## 5. EMT, TME, and Therapeutic Resistance of Tumor Cells

Resistance to therapy is one of the biggest challenges in tumor biology and was initially identified in the early 1990s in breast cancer cells [160]. EMT was implicated in conferring resistance to both conventional therapies such as radiation and chemotherapy and targeted therapies like the estrogen antagonists, Tamoxifen and Fulvestrant or cell cycle inhibitors, each used in specific subtypes of breast cancer. However, in recent years, immunotherapy has gained momentum. Under this section, we will discuss the effects of an EMT-driven protumorigenic TME on different therapeutic options, primarily focusing on chemo and immunotherapy resistance. 

### 5.1. Chemotherapy

The response to chemotherapeutic drugs in breast cancer varies from patient to patient. Various groups have studied the correlation between the degree of response and immune cells present in the TME. Denkert C et al. 2010 showed that tumor-associated lymphocytes are an independent predictor of anthracycline/taxane response in breast cancer patients [161]. It is worth noting that these tumor-infiltrating lymphocytes could also promote EMT in multiple ways [162], further supporting the premise that the response to anthracycline/taxane could be dependent on EMT in tumor cells. In support, a recent study by Salvagno et al. demonstrated that targeting macrophages that are directly linked to EMT in tumors enhances the chemotherapeutic response of spontaneous mammary tumors [163]. In addition, Ladoire et al. observed that prior to neoadjuvant chemotherapy, patients display increased numbers of CD3-, CD8-, and FOXP3-positive cells [164]. However, patients who responded to therapy had significantly fewer FOXP3-positive cells than did nonresponders, in whom FOXP3-positive cells remained high. The authors concluded that high CD8^+^ and low FOXP3^+^ staining predicts a better response to neoadjuvant therapy in breast cancer. In contrast, other studies suggest that TNFα secreted by CD8^+^ cells through sphingosine kinase mediates tamoxifen resistance in MCF7 cells [165,166]. Therefore, correlation of CD8+ cells secreting TNFα and tamoxifen response needs further evaluation.

A potential role for MDSC-mediated increases in CSCs in chemoresistance has also been noted. Specifically, Montero et al. observed that the number of circulating MDSCs in breast cancer patients increase upon Doxorubicin-cyclophosphamide chemotherapy [167]. As MDSCs promote EMT in tumor cells, the consequent increase in CSC-like properties [139,168] could be responsible for decreased efficacy of Doxorubicin-cyclophosphamide in breast cancer patients. Together, these studies suggest that drug resistance to chemotherapy is linked to altered immune cells and cancer cells which needs to be studied in depth for better development of drugs against resistance.

Similar to chemotherapy, several monoclonal antibodies targeting immune cells such as Tregs (CD25 antibody) have shown some success in preclinical models, however, their function as monotherapy in established patient tumors is limited [169]. Moreover, antiangiogenic therapy with antibodies against vascular endothelial growth factor (VEGF) has not proven effective in patients with many tumor types, including breast cancer. VEGF has been shown to involve T cell development and therefore has been suggested to be connected to tumor-induced immune suppression [170]. It is recently shown that such resistance could be due to VEGF-mediated activation of IL-6 involving tumor microenvironment [171]. Trastuzumab, an FDA-approved anti-HER2 antibody, shows a 35% response rate in metastatic breast cancer patients, however, exact mechanisms of action are still unknown. It is believed that Trastuzumab alters signaling activation of immune effector mechanisms. It would be interesting to determine if such resistance is due to involvement of EMT, tumor microenvironment, and immune cells [172]. 

### 5.2. Immunotherapy

TNBC tumors, which lack known hormone receptors, are insensitive to hormone-based therapies and are often resistant to chemo and radiotherapy. However, these tumors are highly immunogenic and therefore immunotherapy for treatment of TNBC may be particularly useful. Checkpoint inhibitors such as PD-1 (Nivolumab, Pembrolizumab), PD-L1 (Atezolizumab, Avelumab), and CTLA4 (Ipilimumab) block the immunomodulatory pathways between tumor cells and immune cells that assist in immune evasion and are currently in clinical trials. PD-L1 expression varies from 20% to 50% in all types of breast cancer subtypes [173,174] and is higher in TNBC patients as compared to non-TNBC [174,175]. Accordingly, variable responses to checkpoint-based therapy could be dependent on expression levels of PD-L1 ligand on tumor cells. PD-L1 expression in EMT-activated breast cancer cells depends on the EMT-TF (ZEB1). Specifically, Noman et al. showed that mutual regulatory loop exists between two processes orchestrated by ZEB1, which functions as a transcriptional repressor of miR-200 that is able to activate the EMT program and as an activator of PD-L1 expression in tumor cells, leading to CD8^+^ T cells immunosuppression [176]. A similar correlation between PD-L1 and ZEB1 expression was found in nonsmall cell lung carcinoma (NSCLC) [177]. In NSCLC, patients with circulating tumor cells (CTCs) positive for PD-L1 were resistant to Nivolumab while those with PD-L1-negative CTCs were responsive [178,179]. Notably, these PD-L1-positive CTCs showed EMT features, identifying EMT as a predictive biomarker for response towards checkpoint inhibitors in breast, NSCLC, and other tumor types. Additionally, these results provide a novel preclinical rationale to explore EMT inhibitors as adjuvants to boost immunotherapeutic responses in subgroups of patients in whom malignant progression is driven by EMT-promoting transcription factors.

Checkpoint inhibitors in combination with chemotherapy drugs increase the rate of complete clinical response in several cancers including breast cancer. Pembrolizumab added to a neoadjuvant regimen consisting of cisplatin and doxorubicin increased the response of patients (NCT01042379) and similar observations have been observed with anti-PDL1 and anti-CTLA4 drugs. A combinatorial approach of more than one checkpoint inhibitor with chemotherapy for treatment of breast cancer patients is currently underway in clinical trial (NCT01928394). Along with designing combinatorial strategies of checkpoint inhibitors and chemotherapeutic drugs, researchers are also trying to inhibit immune cells such as T cells and M1 macrophages along with treatment with checkpoint blockers as potential strategies to overcome resistance to checkpoint inhibitors observed in some patients [180,181]. Together, these studies reveal that immune cells in the TME have a significant impact on the response of patients to different drugs, and suggest that regulation of EMT in tumor cells may provide a way to influence the immune landscape to increase therapeutic response.

## 6. Conclusions

In this review, we have summarized the factors that determine EMT in different breast cancer subtypes and highlighted studies revealing how epithelial cancer cells undergoing EMT modulate the TME to promote tumorigenesis and enhance recruitment of immune cells. Notably, immune cell recruitment further enhances the ability of tumor cells to undergo EMT, thereby assisting in their tumor progression, invasion, and metastasis. Finally, we highlighted how immune cells and stromal components in TME determine the chemotherapeutic and immunotherapeutic response of patients. Resistance to checkpoint inhibitor-based immunotherapy can be answered through investigation of TME components. Blockade of their migration or recruitment into the tumor site may result in better immunotherapeutic response. The recruitment of immune cells into the tumor site is dependent on EMT in tumor cells. Thus future studies identifying novel combination therapies targeting immune cells in TME and tumor cells undergoing EMT will improve prognosis for breast cancer patients. 

**Figure 1:**
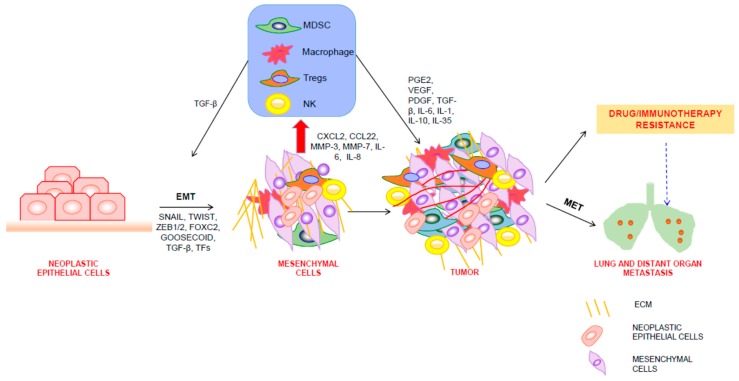
EMT, TME, and therapy. Neoplastic mammary epithelial cells undergoing transcriptional changes in key genes involved in EMT are transformed into mesenchymal cells. These mesenchymal cells secrete extracellular factors responsible for recruitment of immune cells and modulation of ECM. Recruited immune cells provide a proinflammatory milieu for growth of tumors by further secreting growth-promoting and prometastatic cytokines.

In a nutshell, a pictorial representation of the circuit between neoplastic mammary epithelial cells to mesenchymal cells and recruitment of immune cells in TME and its overall impact on therapy is provided in Figure 1. 

## Figures and Tables

**Table 1:** Table shows genes and pathways involved in mediating EMT in different subtypes of breast.

Breast Cancer Subtypes	Genes Involved	Signaling Pathways Involved	References
Luminal A and B	*SLUG*, *BCL2*, *BM1*, *TGF-β*, *TWIST*, *ZEB1/2*, *ETS1*, *VEGFR*	ERα signaling, TGF-β signaling, EGFR-, IGFR-, and MAPK-dependent, MEK–ERK	[36,37,38,39,40,41,42,43,44,45,46]
HER2-positive	*TGF-β*, *TWIST1*, *PTK-6*	TGF-β signaling, AKT signaling, HER2/EGFR signaling	[47,48,49,50,52,53,54]
TNBC (Basal and Claudin-Low)	*TGF-β*, *GLI1*, *SNAIL*, *SLUG*, *TWIST1*, *ZEB1*, *ΔNP63*, *GSK3β*	PI3K/AKT, Notch signaling, Hedgehog signaling, Wnt signaling	[59,63,64,65,66,67,68,69,70,71,72,73,74,75,76,77,78,79]
